# Epidemiology of work-related musculoskeletal disorders in Brazil, 2007-2021

**DOI:** 10.47626/1679-4435-2026-1447

**Published:** 2026-07-23

**Authors:** Guilherme Octhávio Rodrigues da Silva Rosa, Danúbia Hillesheim, Roberto Carlos Ruiz, Fabrício Augusto Menegon, Lizandra da Silva Menegon

**Affiliations:** 1 Universidade Federal de Santa Catarina, Florianópolis, SC, Brazil; 2 Universidade Federal de Santa Catarina, Saúde Pública, Florianópolis, SC, Brazil

**Keywords:** public health, occupational health, cumulative trauma disorders, RSI, WMSD.

## Abstract

**Introduction:**

The intensification of labor exploitation contributes to the occurrence of work-related health conditions. In this context, the increasing prevalence of repetitive strain injuries (RSIs) and work-related musculoskeletal disorders (WMSDs) represents a major concern for the health of Brazilian workers.

**Objectives:**

To describe the epidemiological profile and prevalence rates of RSIs and WMSDs among workers in Brazil based on notification records from the Brazilian Notifiable Diseases Information System (SINAN) between 2007 and 2021.

**Methods:**

A cross-sectional study was conducted using notification data from SINAN and population information obtained from the Brazilian National Household Sample Survey (PNAD), the Demographic Census, and the Continuous PNAD. Sociodemographic variables were described using absolute and relative frequencies. Prevalence rates were estimated according to year, sociodemographic characteristics, macroregions, and Brazil as a whole using Poisson regression.

**Results:**

A total of 102,986 cases were reported during the study period. Over the 15-year period, the states of Piauí, Amapá, Roraima, Acre, and Rondônia each recorded fewer than 4,000 cases. In contrast, São Paulo reported more than 32,000 cases during the same period. The highest prevalence rates were observed in 2017 among women (12.84 per 100,000 workers), self-identified White individuals (8.19 per 100,000 workers), individuals with complete secondary or higher education (4.89 per 100,000 workers), and workers aged 40 to 59 years (10.69 per 100,000 workers).

**Conclusions:**

The highest prevalences of RSIs and WMSDs were observed among women, self-identified White individuals, people with complete secondary or higher education, and workers aged 40 to 59 years. In addition, disparities were identified in both notifications and prevalence rates across Brazilian states and macroregions.

## INTRODUCTION

The intensification of labor exploitation contributes to the occurrence of occupational accidents and work-related illnesses [^1^]. According to Normative Instruction No. 98 of the Brazilian National Institute of Social Security (INSS), published in the Official Gazette of the Union on December 5, 2003, repetitive strain injuries (RSIs) and work-related musculoskeletal disorders (WMSDs) are syndromes characterized by a variety of symptoms, including pain, paresthesia, sensations of heaviness, and fatigue in the upper and lower limbs. Overloading specific body structures as a result of repetitive movements, poor posture, excessive force exertion, and lack of rest breaks may trigger or aggravate these disorders [^2^]. These conditions impose physical and psychological burdens on workers and may progress to temporary or permanent disabilities, as described in Informative Note No. 94 of the Health Surveillance Secretariat of the Ministry of Health, published on July 26, 2019, and Joint Informative Note No. 005 of the Santa Catarina State Department of Health, 2023 [^3^]. In this context, working conditions are directly associated with RSIs and WMSDs, as these conditions may be caused, maintained, or aggravated by the management and organization of work.

RSIs and WMSDs are highly prevalent in different countries and represent a major occupational health issue worldwide [^4^-^9^]. In Europe, the European Agency for Safety and Health at Work (EU-OSHA) estimated a prevalence of 23,000 cases per 100,000 workers of self-reported work-related musculoskeletal symptoms among European Union member states (EU27) in 2007 [^4^]. In Brazil, data from the National Health Survey (PNS) indicated a prevalence of 2,400 cases per 100,000 workers in 2013 [^6^], increasing to 2,500 cases per 100,000 workers in 2019 [^7^]. These findings suggest important differences between the national and international scenarios.

Santana et al. [^1^] demonstrated, in a literature review covering the period from 1999 to 2004, that at least one-third of fatal and nonfatal occupational accidents in Porto Alegre were underreported. Among fatal cases, underreporting reached 81% between 1992 and 1993 [^9^]. Conversely, other authors identified improvements in the quality of data from the Brazilian Notifiable Diseases Information System (SINAN), classifying it as ranging from fair to excellent between 2007 and 2011 in the state of Minas Gerais [^10^]. This scenario raises questions regarding the true magnitude of work-related health conditions in Brazil, as limitations in information systems may compromise the identification of all cases. Consequently, a substantial portion of workers’ suffering and illness may remain unrecognized. This heterogeneity in reporting suggests the possibility of underreporting of RSI and WMSD cases in SINAN.

Therefore, this study aimed to describe the epidemiological profile and prevalence rates of RSIs and WMSDs among workers in Brazil based on SINAN notification data from 2007 to 2021.

## METHODS

This was a cross-sectional, epidemiological study that used secondary data from SINAN, obtained from notification forms of workers affected by RSIs and WMSDs between 2007 and 2021. Data were accessed through the website of the Collaborating Center for Surveillance of Worker Health Conditions of the Institute of Collective Health at Universidade Federal da Bahia (http://www.ccvisat.ufba.br/sinan-2/) and were extracted on March 30, 2022.

The selected sociodemographic variables were sex (female, male, and unknown), race/skin color (White and non-White, with Black, Brown, Asian, and Indigenous individuals grouped together because of the high proportion of missing records), educational level (up to incomplete elementary education; complete elementary education to incomplete secondary education; complete secondary education and higher education; and unknown), age group (14-24 years, 25-39 years, 40-59 years, 60 years or older, and unknown), year of notification (2007-2021), Brazilian states, Brazilian macroregions (North, Northeast, Southeast, South, and Central-West), Brazil, and issuance of the Work Accident Report (CAT, in Portuguese) (yes, no, and unknown).

The variables were described using absolute and relative frequencies. Prevalence rates were estimated using Poisson regression models and expressed per 100,000 economically active workers. For rate calculations, the numerator consisted of RSI and WMSD notifications recorded in SINAN.

The denominator was obtained from three population-based data sources related to the employed population. Although these sources present methodological differences and specific limitations, they were considered the most appropriate for estimating the Brazilian working population throughout the study period. Data for 2007-2009 and 2011-2015 were obtained from the third quarter of the Brazilian National Household Sample Survey (PNAD). Data for 2010 were extracted from the Demographic Census. For the period from 2016 to 2021, data from the third quarter of the Continuous PNAD were used.

Self-identified Asian and Indigenous populations were not available in the Continuous PNAD between 2016 and 2021. Therefore, for these categories, the estimated population for 2015 was used as a proxy for subsequent years. The use of third-quarter data from both PNAD and the Continuous PNAD was justified because this was the only period with consistently available information across all years included in the analysis. Additionally, Asian, Indigenous, Brown, and Black individuals were grouped into the “non-White” category to reduce the impact of missing data related to the race/skin color variable and to improve the stability of the estimates.

Data organization and analysis were performed using Stata version 14 (StataCorp LLC, College Station, T.X., USA) and Microsoft 365 (Microsoft Corporation, Redmond, W.A., USA).

As this study was based on publicly available secondary data that had been previously anonymized and did not allow identification of individuals, ethical review by a human research ethics committee was waived in accordance with Resolution No. 510 of April 7, 2016, of the Brazilian National Health Council.

## RESULTS

Between 2007 and 2021, SINAN recorded 102,986 notifications of RSIs and WMSDs. The annual number of notifications ranged from 3,228 to 9,885 cases, showing an upward trend between 2007 and 2017, followed by a decline through 2020. The most frequently reported groups were women (52.12%), self-identified White individuals (40.04%), workers with complete secondary or higher education (41.20%), and those aged 40 to 59 years (52.68%). Issuance of a CAT was recorded in 41.49% of notifications. Among the variables analyzed, the highest proportions of missing data were observed for CAT issuance (28.43%), educational level (25.91%), and race/skin color (24.53%) (Table 1).

**Table 1:** Description of the sociodemographic and occupational characteristics of workers with RSI and WMSD notifications, Brazil, 2007-2021 [^11^]

Variables	Total	
	n	%
Sex		
Female	53,680	52.12
Male	49,297	47.87
Unknown	9	0.01
Race/Skin color		
White	41,240	40.04
Black	35,533	34.50
Asian	710	0.69
Indigenous	242	0.23
Unknown	25,261	24.53
Educational level		
Up to incomplete elementary education	20,143	19.56
Complete elementary education to incomplete secondary education	13,727	13.33
Complete secondary education to higher education	42,432	41.20
Unknown or not applicable	26,684	25.91
Age group, years		
14-24	5,206	5.06
25-39	39,237	38.10
40-59	54,254	52.68
≥ 60	3,744	3.64
Unknown	545	0.53
CAT issuance		
Yes	42,730	41.49
No	30,981	30.08
Unknown	29,275	28.43
Year		
2007	3,228	3.13
2008	3,474	3.37
2009	4,713	4.58
2010	5,951	5.78
2011	7,205	7.00
2012	8,343	8.10
2013	8,134	7.90
2014	8,341	8.10
2015	9,408	9.14
2016	9,160	8.89
2017	9,885	9.60
2018	8,774	8.52
2019	6,772	6.58
2020	4,340	4.21
2021	5,258	5.11
Total	102,986	100

Source: Notifiable Diseases Information System. Brazil, 2007-2021.

CAT: Work Accident Report; RSI: repetitive strain injury; WMSD: work-related musculoskeletal disorder.

**Figure 1:**
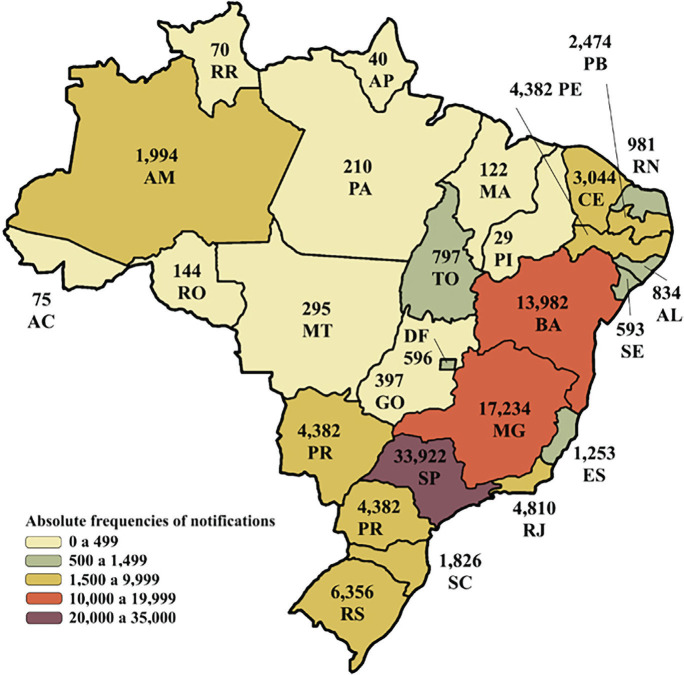
Absolute frequencies of notifications of repetitive strain injuries and work-related musculoskeletal disorders according to Brazilian States. Brazil, 2007-2021 [^11^].

Source: Notifiable Diseases Information System. Brazil, 2007-2021.

Regarding Brazilian states, only three recorded more than 10,000 RSI and WMSD notifications during the study period: São Paulo (n = 33,922), Minas Gerais (n = 17,324), and Bahia (n = 13,982). In contrast, six states recorded fewer than 150 notifications between 2007 and 2021: Piauí (n = 29), Amapá (n = 40), Roraima (n = 70), Acre (n = 75), Maranhão (n = 122), and Rondônia (n = 144) (Figure 1).

**Figure 2:**
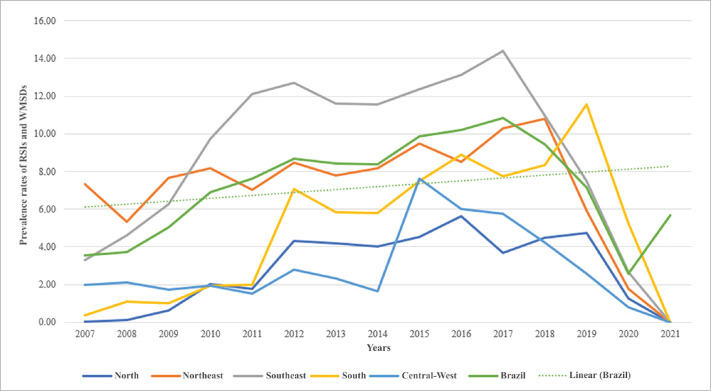
Prevalence rates of repetitive strain injuries and work-related musculoskeletal disorders per 100,000 workers according to Brazilian macroregions and Brazil. Brazil, 2007-2021 [^11^].

Source: Notifiable Diseases Information System. Brazil, 2007-2021.

The prevalence of RSIs and WMSDs in Brazil ranged from 2.56 to 10.83 cases per 100,000 workers over the 15-year period analyzed. Across macroregions, rates ranged from 0.05 to 14.40 cases per 100,000 workers. The Southeast Region presented the highest prevalences throughout the study period, whereas the Central-West Region recorded the lowest rates. In 2015, the prevalence observed in the Southeast was 291% higher than that of the North Region. In 2017, the North Region presented a prevalence of approximately 0.05 cases per 100,000 workers. In the same year, the prevalence observed in the Northeast was 145.6% higher than that recorded in the North (Figure 2).

Women presented higher prevalence rates of RSIs and WMSDs than men in all years analyzed, except in 2010, when the rates were 6.26 and 7.74 cases per 100,000 workers, respectively. The highest rate among women was observed in 2017, reaching 12.84 cases per 100,000 workers, which was 37% higher than the rate observed among men in the same year.

Regarding race/skin color, self-identified White workers generally presented higher prevalences of RSIs and WMSDs. In 2015, the prevalence in this group was 8.29 cases per 100,000 workers, which was 42% higher than that observed among non-White workers. However, in 2016, this pattern was reversed, with a prevalence of 11.55 cases per 100,000 workers among non-White workers, corresponding to a value 34% higher than that recorded among White workers.

Regarding educational level, in 2015, workers with complete secondary or higher education presented a prevalence of 7.78 cases per 100,000 workers, which was 69% higher than that observed among workers with up to incomplete elementary education. Throughout most of the study period, this group exhibited higher prevalences of RSIs and WMSDs. In addition, workers aged 40 to 59 years presented the highest prevalences in all years analyzed. In 2015, the prevalence in this group was 316% higher than that observed among workers aged 14 to 24 years (Table 2).

**Table 2:** Prevalence rates of RSIs and WMSDs per 100,000 workers according to sociodemographic characteristics. Brazil, 2007 2021 [^11^]

Variables	2007	2008	2009	2010	2011	2012	2013	2014	2015	2016	2017	2018	2019	2020	2021
Sex															
Female	4.18	4.76	6.22	6.26	9.66	10.85	10.17	9.91	11.64	12.25	12.84	11.93	9.28	6.46	7.08
Male	3.09	2.95	4.13	7.74	6.10	7.08	7.11	7.23	8.55	8.70	9.33	7.58	5.54	4.32	4.63
Race/Skin color															
White	2.02	2.67	3.92	5.53	6.00	7.30	7.25	7.26	8.29	8.59	8.19	8.46	7.23	4.68	5.32
Non-White	3.46	3.00	3.90	2.50	4.69	5.69	4.96	5.17	5.82	11.55	6.82	7.30	5.09	3.40	4.06
Educational level															
Up to incomplete elementary education	2.16	2.55	3.16	4.71	5.38	5.68	4.61	3.86	4.60	2.34	2.44	2.70	2.48	1.52	1.81
Complete elementary education to incomplete secondary education	3.58	3.13	3.94	5.59	6.26	6.90	6.58	6.16	6.39	3.49	3.76	4.38	3.27	2.38	2.90
Complete secondary education to higher education	3.07	2.94	4.66	6.20	6.45	7.59	7.28	7.19	7.78	5.51	4.89	5.09	3.72	2.07	2.49
Age group, years															
14-24	1.08	0.72	1.58	1.98	2.03	3.31	2.83	2.97	3.18	0.90	1.18	1.12	0.70	0.57	0.67
25-39	3.82	3.84	5.39	7.16	7.99	9.30	9.20	9.39	10.25	7.05	7.02	6.09	4.30	2.76	3.00
40-59	5.37	5.67	7.28	10.22	11.03	1.19	11.38	11.00	13.23	9.83	10.69	9.37	7.48	4.57	5.66
≥ 60	0.83	0.67	1.35	2.22	2.43	2.70	2.74	2.92	4.43	1.42	1.72	1.53	1.35	0.91	1.25
Brazil															
Overall	3.55	3.72	5.03	6.89	7.60	8.68	8.42	8.39	9.86	10.20	10.83	9.44	7.15	2.56	5.66

Source: Notifiable Diseases Information System. Brazil, 2007-2021.

RSI: repetitive strain injury; WMSD: work-related musculoskeletal disorder.

## DISCUSSION

This study identified 102,986 notifications of RSIs and WMSDs in Brazil between 2007 and 2021, with a 205% increase in the prevalence rate during the study period. The highest prevalences were observed among women, self-identified White individuals, workers with complete secondary or higher education, and individuals aged 40 to 59 years. In addition, more than half of the records either lacked CAT issuance or contained missing information regarding this variable.

There was substantial heterogeneity in the distribution of notifications across Brazilian states. São Paulo accounted for the highest number of notifications throughout the 15-year period analyzed. In contrast, Piauí, Amapá, Roraima, Acre, and Rondônia collectively accounted for only 358 notifications during the study period, a figure substantially lower than that reported in São Paulo alone. These findings suggest the existence of areas with limited capacity for case detection and reporting, a phenomenon frequently described in literature as the presence of silent municipalities.

The implementation of SINAN-Net in 2007 and the inclusion of RSIs and WMSDs in the list of universally notifiable conditions in 2014 may have contributed to the increase in notifications observed between 2007 and 2017 [^12^]. However, these factors do not appear to fully explain the differences observed among Brazilian states. Part of this heterogeneity may be related to structural inequalities in occupational health surveillance, including difficulties in case diagnosis, limitations in the training of health professionals for reporting purposes, insufficient issuance of CATs, deficiencies in data quality, and a lack of standardization among the population databases used for epidemiological estimates [^13^].

The reduction in notifications observed after 2017 may be associated with multiple factors. Among them are the changes introduced in Primary Health Care following the revision of the Brazilian National Primary Care Policy, which modified structural aspects of the Family Health Strategy (FHS). Garcia & Socal [^14^] argued that these changes represented a setback in the national care strategy, with potential impacts on population access to health care services. However, Bastos-Ramos et al. [^13^] did not identify an association between Family Health coverage and percentage changes in occupational accident notifications recorded in SINAN between 2008 and 2009. This situation may have affected occupational health actions within primary health care through the end of the study period in 2021. In this context, the weakening of the FHS may have contributed to a growing disconnect between primary care policies and occupational health surveillance activities across states, reducing the time available for reporting health conditions, issuing CATs, and developing intersectoral collaborations.

The limitations observed in the coverage of occupational health surveillance activities throughout the national territory raise concerns regarding the reliability of SINAN records, both in terms of data quality and notification completeness.

The prevalence rates of RSIs and WMSDs identified in this study were lower than those reported in national and international investigations. In Brazil, the 2013 PNS, conducted with 60,202 employed workers aged 18 years or older, estimated a prevalence of 2,400 cases per 100,000 workers [^7^]. In 2019, the PNS, based on a sample of 94,114 employed workers, estimated a prevalence of 2,500 cases per 100,000 workers [^8^].

However, comparisons between the findings of this study and those of the PNS should be interpreted with caution because the databases differ substantially in terms of the populations studied, methods of data collection, and case definitions. Despite these limitations, the scarcity of studies simultaneously using SINAN data and worker population estimates justifies such comparisons, even if only on an exploratory basis.

Internationally, a study conducted in the United States using data from the National Health Interview Survey, involving 17,524 currently employed or recently employed adults, identified a clinical and diagnostic prevalence of carpal tunnel syndrome of 6,700 cases per 100,000 workers, with 67.1% of cases attributed to work-related factors [^15^]. In China, a study conducted in 2019 involving 1,415 workers from six industries in Beijing found a prevalence of 35,000 RSI and WMSD cases per 100,000 workers (35%) [^16^].

In 2007, the EU-OSHA reported that approximately one-quarter of workers in the European Union (EU27) experienced work-related musculoskeletal symptoms, assessed using the Nordic Musculoskeletal Questionnaire (NMQ) [^4^]. Subsequently, between 2010 and 2015, the same agency estimated that at least three out of every five workers in the European Union (EU28) reported work-related musculoskeletal symptoms during the previous 12 months [^5^].

Comparing the prevalence of RSIs and WMSDs identified in this study with the PNS estimates (2.4%-2.5%) and the highest prevalence observed in Brazil in 2017 (10.83 cases per 100,000 workers), it is possible to estimate underreporting of RSI and WMSD prevalence ranging from 99.76% to 99.77%. This finding may further support the hypothesis of underreporting in Brazil since even countries with higher levels of development and greater investment in health care report prevalences of RSIs and WMSDs that exceed those observed in the present study.

During the study period, women were more affected by RSIs and WMSDs than men. In 2017, the prevalence was 37% higher among women, a finding consistent with studies conducted in China, Portugal, and Brazil [^15^-^20^]. A study conducted in 1994 in Taiwan involving 22,475 workers found a higher prevalence of RSIs and WMSDs among women compared with men (39.5% vs. 35.2%) [^17^]. Another study conducted between 2009 and 2010 in the city of Puning across seven public schools showed that women had a higher prevalence of neck and/or shoulder pain than men (51.7% vs. 42.7%; p < 0.05) [^18^]. In Portugal, a study conducted in 2019 involving 705 endoscopists from the Portuguese Society of Gastroenterology found a prevalence rate of RSIs and WMSDs that was 52% higher among women than among men [^19^].

In Brazil, several studies have reinforced the higher rates of RSIs and WMSDs among women. In a study conducted in Salvador in 2010, 577 workers from the plastics industry completed the Nordic Musculoskeletal Questionnaire (NMQ), and the prevalence of upper-limb RSIs and WMSDs was 35,000 cases per 100,000 women compared with 12,000 cases per 100,000 men [^15^]. In another study conducted in 2012 in São Miguel do Iguaçu, 201 slaughterhouse workers participated in an analysis of handgrip strength. Women were found to have lower handgrip strength and higher prevalences of work-related pain in the neck (25,000 per 100,000 workers), shoulders (21,500 per 100,000 workers), arms (48,700 per 100,000 workers), spine (48,000 per 100,000 workers), and hands and wrists (48,800 per 100,000 workers) compared with men [^16^].

These studies demonstrate the greater vulnerability of women to RSIs and WMSDs but do not fully explain the higher prevalence observed compared with men. Socio-occupational conditions characterized by work intensification and precarious employment, combined with the sexual and social division of labor, including second and third shifts related to childcare and domestic responsibilities, create scenarios of poorer working conditions and greater occupational hazards for women. Although biological factors should be considered, they do not constitute the primary cause of illness among women. Thus, women are systematically more exposed to risk factors in both occupational and domestic environments than men [^20^].

White workers accounted for the highest absolute number of RSI and WMSD notifications and, during most of the study period, exhibited the highest prevalence rates. However, in 2007, 2008, and 2016, non-White workers presented higher prevalence rates. Approximately 25% of records for the race/skin color variable were missing, and the absence of this information may mask vulnerabilities among non-White workers.

This potential vulnerability is frequently observed among non-White populations, as this group continues to be disproportionately represented in precarious, repetitive occupations with high physical and mental demands. Santos et al. reported that incomplete records hinder the understanding of the occupational reality of the Black population, potentially obscuring important inequalities and generating discrepancies between official statistics and data produced by social movements [^21^].

Although workers with lower educational attainment would be expected to have a stronger association with RSIs and WMSDs because they are more frequently engaged in physically demanding activities, a study conducted between 2009 and 2019 found higher rates of these conditions among workers with complete secondary and higher education. This finding was attributed primarily to the shift in work patterns from factory-based activities to office-based work involving continuous use of computers and computer mice [^22^,^23^]. A systematic review published in 2007 identified that longer durations of computer and mouse use increased vulnerability to and incidence of RSIs and WMSDs [^21^]. Griffiths et al., in a cross-sectional study conducted over a 12-month period in the Australian public sector, suggested that administrative and secretarial workers were exposed to a higher frequency of repetitive movements, representing another important risk factor [^23^].

Workers aged 40-59 years presented the highest prevalence rates of RSIs and WMSDs in every year analyzed. Prevalence increased until 2015 (13.23 per 100,000 workers), followed by a gradual decline through 2020. In 2015, this population group exhibited a prevalence of RSIs and WMSDs that was 316% higher than that observed among workers aged 14-25 years. One possible explanation for this disparity is that workers aged 40-59 years generally accumulate prolonged exposure to occupational risk factors throughout their professional careers [^24^].

CATs were issued in 42% of RSI and WMSD notifications (n = 42,730). According to Cordeiro et al., the low number of CAT issuances reflects the poor quality of information regarding occupational accidents and work-related illnesses in Brazil [^25^]. Filgueiras & Carvalho emphasized that failure to report occupational conditions contributes to reducing employers’ costs because, by omitting notification, employers avoid initiating procedures for the granting of occupational injury benefits by INSS. This directly or indirectly shifts responsibility for illness onto workers themselves, exempting employers from accountability [^26^].

Even after the introduction of the Social Security Epidemiological Technical Nexus in 2007, Filgueiras & Carvalho [^26^] suggested that this measure was insufficient to combat the concealment of occupational accidents identified by the INSS. Data from the Brazilian Institute of Geography and Statistics and the Ministry of Health, derived from the 2013 PNS, showed that approximately 4.9 million individuals aged 18 years or older experienced occupational accidents in Brazil, a figure approximately 7 times higher than that captured by the INSS. Furthermore, INSS data from 2007 to 2013 regarding dorsalgia, shoulder injuries, synovitis, and tenosynovitis demonstrated that these occupational conditions resulted in more than twice as many benefit approvals as the number of CATs issued. These findings reinforce that the INSS identifies more occupational diseases in the absence of CATs than through employer notifications, highlighting a tendency among employers to conceal RSI and WMSD cases [^26^].

Although part of the population data used to calculate prevalence rates was missing, underreported, or subject to proportional differences, the analyses demonstrated a good capacity to identify potential disparities in RSI and WMSD notification and prevalence rates across population groups and regions. In this regard, the findings support the suggestion of inefficient epidemiological surveillance policies in several Brazilian states.

Finally, it is important to acknowledge the limitations and challenges of this study. Adaptations and exclusions were necessary when using the population denominators employed to calculate prevalence rates throughout the study period, including the generalization of Indigenous and Asian populations, the absence of population data for 14-year-old individuals in the PNAD, the use of first-quarter epidemiological estimates, and the unavailability of macroregional data for 2021. In addition, this study focused exclusively on the analysis of SINAN data and did not include other databases and information systems that also record RSI and WMSD notifications.

## CONCLUSIONS

This study revealed a 205% increase in the prevalence of RSIs and WMSDs in Brazil between 2007 and 2021. The population groups with the highest prevalences were women, White workers, individuals with complete secondary or higher education, and those aged 40 to 59 years. Disparities in notifications were also observed across Brazilian states. While São Paulo recorded the highest number of cases (n = 33,922), Piauí reported only 29 notifications throughout the entire study period.

The notification rates identified in this study were substantially lower than those reported in national and international investigations, indicating that RSI and WMSD cases in Brazil are highly underreported within the health information systems of the Unified Health System (SUS). This scenario represents a significant barrier to the implementation of the National Policy for Workers’ Health.

This study contributes to the planning of occupational health surveillance actions throughout the national territory by highlighting the urgent need to strengthen recognition of the role of work in determining health-disease processes among SUS health care teams. Therefore, combating underreporting and improving the quality of records appear to be important strategies for developing more reliable indicators regarding the profile of RSIs and WMSDs among Brazilian workers.

This article is based on the undergraduate thesis of Guilherme de Octhávio Rodrigues da Silva Rosa, defended in 2023 at Universidade Federal de Santa Catarina, available at: https://repositorio.ufsc.br/handle/123456789/255978


## Data Availability

Upon publication, the data will be made available by the authors upon request.

## References

[1] Santana V, Nobre L, Waldvogel BC. (2005). Acidentes de trabalho no Brasil entre 1994 e 2004: uma
revisão. Cienc Saude Colet.

[2] Brasil (2003). Instrução Normativa DC/INSS nº 98, de 5 de dezembro de
2003.

[3] Santa Catarina (2023). Nota Informativa Conjunta nº 005/2023.

[4] Podniece Z, Pinder A, Yeomans L, van den Heuvel S, Blatter B, Verjans M (2007). Work-related musculoskeletal disorders: back to work report.

[5] de Kok J, Vroonhof P, Snijders J, Roullius G, Clarke M, Peereboom K (2019). Work-related musculoskeletal disorders: prevalence, costs and
demographics in the EU.

[6] Instituto Brasileiro de Geografia e Estatística, org (2015). Pesquisa nacional de saúde, 2013: acesso e
utilização dos serviços de saúde, acidentes e
violências: Brasil, grandes regiões e unidades da
Federação.

[7] Instituto Brasileiro de Geografia e Estatística (2021). Pesquisa nacional de saúde, 2019: ciclos de vida: Brasil.

[8] Luckhaupt SE, Dahlhamer JM, Ward BW, Sweeney MH, Sestito JP, Calvert GM. (2013). Prevalence and work-relatedness of carpal tunnel syndrome in the
working population, United States, 2010 National Health Interview
Survey. Am J Ind Med.

[9] Wang T, Zhao YL, Hao LX, Jia JG. (2019). Prevalence of musculoskeletal symptoms among industrial employees
in a modern industrial region in Beijing, China. Chin Med J (Engl).

[10] Suprinyak FH, Menegolla IA. (2023). Avaliação do sistema de vigilância em
saúde do trabalhador relacionada aos acidentes de trabalho, antes e
após a implantação do software de linkage Sentinela, em
Porto Alegre, 2018-2021. Rev Eletron Comun Inf Inov Saude.

[11] Silva GOR. (2023). LER/DORT são subnotificadas no Brasil: análise dos dados
do SINAN entre 2007 e 2021 [trabalho de conclusão de curso].

[12] Galdino A, Santana VS, Ferrite S. (2017). Qualidade do registro de dados sobre acidentes de trabalho fatais
no Brasil. Rev Saude Publica.

[13] Bastos-Ramos TP, Santana VS, Ferrite S. (2015). Estratégia Saúde da Família e
notificações de acidentes de trabalho, Brasil,
2007-2011. Epidemiol Serv Saude.

[14] Garcia FL, Socal M. (2022). Impacts of the 2017 Brazilian National Primary Care Policy on
public primary health care in Rio de Janeiro, Brazil. Cad Saude Publica.

[15] Fernandes RCP, Assuncao AA, Silvany-Neto AM, Carvalho FM. (2010). Musculoskeletal disorders among workers in plastic manufacturing
plants. Rev Bras Epidemiol.

[16] Reis P, Moro A, Merino E, Vilagra J. (2012). Influence of gender on the prevalence of RSI/WRULD in
meat-packing plants. Work.

[17] Guo HR, Chang YC, Yeh WY, Chen CW, Guo YL. (2004). Prevalence of musculoskeletal disorder among workers in Taiwan: a
nationwide study. J Occup Health.

[18] Yue P, Liu F, Li L. (2012). Neck/shoulder pain and low back pain among school teachers in
China: prevalence and risk factors. BMC Public Health.

[19] Morais R, Vilas-Boas F, Pereira P, Lopes P, Simoes C, Dantas E (2020). Prevalence, risk factors and global impact of musculoskeletal
injuries among endoscopists: a nationwide European study. Endosc Int Open.

[20] Wijnhoven HAH, de Vet HCW, Picavet HSJ. (2006). Prevalence of musculoskeletal disorders is systematically higher
in women than in men. Clin J Pain.

[21] Santos RV, Bastos JL, Kaingang JD, Batista LE. (2022). Cabem recomendações para usos de “raça” nas
publicações em saúde? Um enfático “sim”,
inclusive pelas implicações para as práticas
antirracistas. Cad Saude Publica.

[22] IJmker S, Huysmans MA, Blatter BM, van der Beek AJ, van Mechelen W, Bongers PM. (2007). Should office workers spend fewer hours at their computer? A
systematic review of the literature. Occup Environ Med.

[23] Griffiths KL, Mackey MG, Adamson BJ, Pepper KL. (2012). Prevalence and risk factors for musculoskeletal symptoms with
computer based work across occupations. Work.

[24] Palmer KT, Goodson N. (2015). Ageing, musculoskeletal health and work. Best Pract Res Clin Rheumatol.

[25] Cordeiro R, Sakate M, Clemente APG, Diniz CS, Donalisio MR. (2005). Subnotificação de acidentes do trabalho não
fatais em Botucatu, SP, 2002. Rev Saude Publica.

[26] Filgueiras V, Carvalho SA., Filgueiras V (2017). Saúde e segurança do trabalho no Brasil.

